# Nanocoral-like Polyaniline-Modified Graphene-Based Electrochemical Paper-Based Analytical Device for a Portable Electrochemical Sensor for Xylazine Detection

**DOI:** 10.1021/acsomega.2c00295

**Published:** 2022-04-12

**Authors:** Kasrin Saisahas, Asamee Soleh, Kiattisak Promsuwan, Jenjira Saichanapan, Apichai Phonchai, Nabeesathul Sumayya Mohamed Sadiq, Way Koon Teoh, Kah Haw Chang, Ahmad Fahmi Lim Abdullah, Warakorn Limbut

**Affiliations:** † Forensic Science Programme, School of Health Sciences, Universiti Sains Malaysia, Kubang Kerian, Kelantan 16150, Malaysia; ‡ Center of Excellence for Trace Analysis and Biosensors (TAB-CoE), Prince of Songkla University, Hat Yai, Songkhla 90110, Thailand; § Center of Excellence for Innovation in Chemistry, Faculty of Science, Prince of Songkla University, Hat Yai, Songkhla 90110, Thailand; ∥ Division of Physical Science, Faculty of Science, Prince of Songkla University, Hat Yai, Songkhla 90110, Thailand; ⊥ Division of Health and Applied Sciences, Faculty of Science, Prince of Songkla University, Hat Yai, Songkhla 90110, Thailand; # Forensic Science Innovation and Service Center, Prince of Songkla University, Hat Yai, Songkhla 90110, Thailand

## Abstract

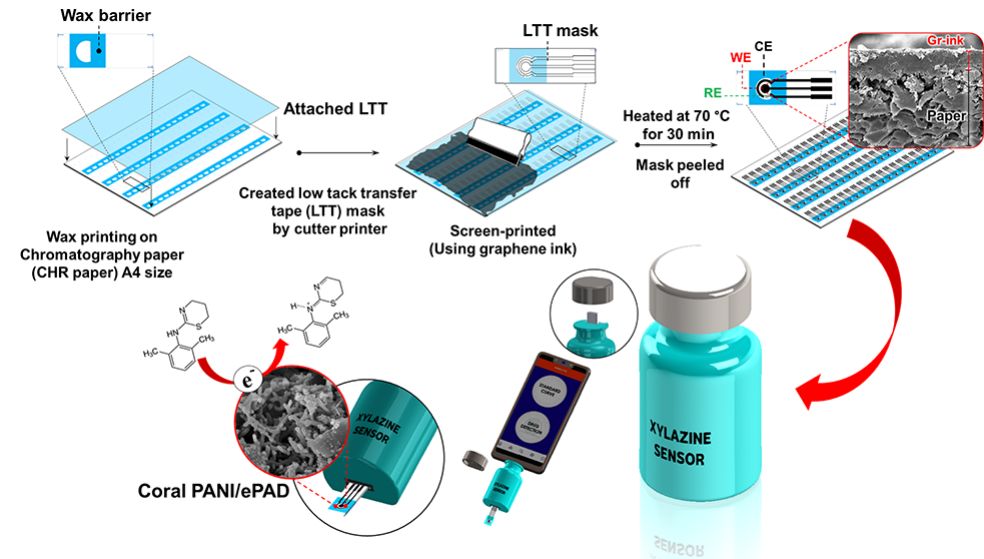

A portable electrochemical device for xylazine detection is presented for the first time. An electrochemical paper-based analytical device (ePAD) was integrated with a smartphone. The fabrication of the ePAD involved wax printing, low-tack transfer tape, and cutting and screen-printing techniques. Graphene ink was coated on the substrate and modified with nanocoral-like polyaniline, providing an electron transfer medium with a larger effective surface area that promoted charge transfer. The conductive ink on the ePAD presented a thickness of 25.0 ± 0.9 μm for an effective surface area of 0.374 cm^2^. This sensor was then tested directly on xylazine using differential pulse voltammetry. Two linear responses were obtained: from 0.2 to 5 μg mL^–1^ and from 5 to 100 μg mL^–1^. The detection limit was 0.06 μg mL^–1^. Reproducibility was tested on 10 preparations. The relative standard deviation was less than 5%. The applicability of the sensor was evaluated with beverage samples spiked with trace xylazine. Recoveries ranged from 84 ± 4 to 105 ± 2%. The developed sensor demonstrated excellent accuracy in the detection of trace xylazine. It would be possible to develop the portable system to detect various illicit drugs to aid forensic investigations.

## Introduction

Drug abuse is a common public health problem, threatening body, life, and property. The misuse of veterinary drugs has been recently reported, and one specific, non-opioid, sedative drug conventionally used for analgesia, hypnosis, and muscle relaxation^1^ has been highlighted, namely, xylazine. This veterinary drug has been used by criminals in robbery and rape cases due to its colorless, odorless, and tasteless nature. A victim might not be able to detect the drug in a spiked drink.

A strong depressant on the human central nervous system, xylazine [*N*-(2,6-dimethyl phenyl)-5,6-dihydro-4*H*-1,3-thiazin-2-amine] was initially synthesized for use in the treatment of hypertension.^2,3^ It induced bradycardia, hypotension, and transient hyperglycemia.^4^ Due to its effects and implications, the Food and Drug Administration restricted xylazine for human use.^3,5^ Currently, xylazine can only be used for analgesic, anesthetic, and sedative purposes in cattle, sheep, goats, horses, cats, and primates. However, it has been illegally traded and used in crime.^6,7^ In humans, xylazine causes drowsiness, diarrhea, muscle relaxation, and pain relief.^3,8^ It primarily affects the central nervous system, and depending on the dosage, it causes exhaustion, sleepiness, muscle weakness, and a reduction in the respiratory rate.^8−10^ Generally, xylazine is metabolized, absorbed, and excreted rapidly.^11^ Symptoms due to the administration of xylazine appear within minutes and can last up to 4 h.^3,12^ Xylazine has been reported to cause initial hypertension, which then decreases, stabilizes, and leads to arrhythmia.^9^ The effects of xylazine could be attenuated, blocked, and reversed with the α2-adrenergic antagonist yohimbine.^13^ In the past decade, xylazine became a popular recreational drug worldwide^3,14^ and has been widely used to adulterate illicit drugs such as cocaine, heroin, and speedball (a mixture of cocaine and heroin).^15^ However, the toxic effects of xylazine in combination with heroin and/or cocaine or other drugs in humans have remained unexplored due to the restriction of its administration to humans.^15,16^

The analytical techniques to determine xylazine proposed in the literature have included high-performance liquid chromatography with ultraviolet absorbance detection,^17^ gas chromatography coupled with mass spectrometry,^6^ and liquid chromatography with mass spectrometry.^11,18^ Although these techniques are very sensitive and selective, they are time-consuming and require costly instrumentation, sophisticated analyses, and specialized operators. For that reason, electrochemical methods have gained increasing attention for their simplicity, rapidity, sensitivity, cost-effectiveness, and suitability for field analysis. However, current research on the electrochemical detection of xylazine is rare. A glassy carbon electrode (GCE)^19^ and a modified carbon paste electrode^20^ were developed for the electrochemical determination of xylazine. However, the peak oxidation of xylazine reported in these works was observed at high potentials (0.85 and 1.00 V, respectively), and it also had limited practicality for on-site analysis. Motivated by the above limitations, our group previously developed a screen-printed carbon electrode (SPCE) modified with graphene nanoplatelets for on-site analysis that could detect xylazine oxidation at a potential of 0.73 V.^21^ However, the instruments were still quite large and the electrode was expensive. Therefore, we developed and designed a smaller, portable electrochemical device that is more convenient and practical to use.

Electrochemical paper-based analytical devices (ePADs) have great potential for on-site analysis and cost-effective. These devices utilize paper as a substrate for analytical measurements. Their low cost, light weight, flexibility, portability, and suitability for large-scale production make them useful in forensic applications, particularly in resource-constrained countries.^22,23^ Also, the graphene ink used to create the three-electrode system on the paper substrate exhibits excellent electrical conductivity, a high specific surface area, thermal stability, and interesting mechanical properties.^24^ Our previous study highlighted the benefits of graphene, where π···π interactions between aromatic molecules of xylazine and graphene greatly increased adsorption in the pre-concentration step of electrochemical measurement.^21^ Nanostructures of very highly conductive polymers such as polyaniline (PANI),^25−27^ poly pyrrole,^28^ poly(3,4-ethylenedioxythiophene),^29^ and their composites have already been used as electrode surface modifiers. In this work, PANI was chosen to improve the performance of the electrochemical sensor. The synthesis procedure of PANI was easy, and it was highly conductive as well as electrochemically and environmentally stable.^25,26^

**Scheme 1:**
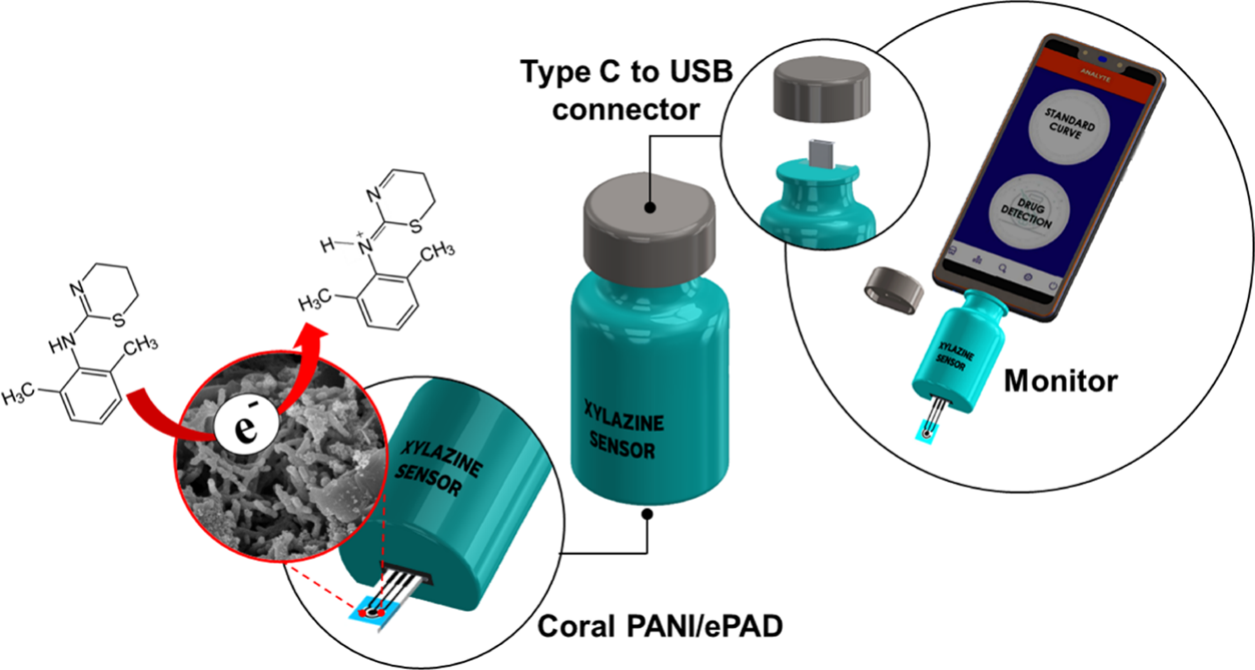
Portable Electrochemical Sensor for Xylazine

The aim of the present work is to establish a novel strategy to determine xylazine using an ePAD based on graphene ink modified with PANI. A small, convenient, and practical portable electrochemical sensor is proposed, as illustrated in Scheme 1. The device resembles a USB drive and connects to a smartphone to control the analytical procedure and display the results. It can support a wide variety of users and does not require a lot of analytical skill. It is hoped that this easy-to-use portable device could enable on-site analysis and direct detection of xylazine.

## Experimental Section

### Reagents and Apparatus

Xylazine hydrochloride standard was purchased from U.S. Pharmacopeia (Rockville, MD). Aniline monomer (ANI, 99%, Sigma-Aldrich, USA), hydrochloric acid (HCl, 37%, Merck, Germany), *N*,*N*-dimethylformamide (DMF, Ajax, Australia), acetic acid (100%, Merck, Germany), boric acid (Ajax, Australia), and phosphoric acid (85.8%, JT Baker, USA) were used as received. Chemicals for interference testing and other substances were obtained from Sigma-Aldrich (St. Louis, USA). Britton–Robinson (BR) buffer was prepared based on a previously reported procedure.^30^ All chemicals used were prepared with deionized (DI) water with a resistivity of 18.2 MΩ cm (Barnstead EasyPure II water purification system, Thermo Scientific, USA).

Chromatography paper (CHR paper, Whatman grade 1 CHR, Cat no. 3001-917) was used to construct the ePAD. Low-tack transfer tape (LTT, Fushun Sticker) was purchased from a local market store. Graphene ink (C2131121D3) and Ag/AgCl ink (C2090225P7) were purchased from Gwent Electronic Materials Co., Ltd. (United Kingdom). A wax printer (Xerox ColorQube 8570, Xerox, USA) was used to create wax barriers. A printer/cutter (Silhouette Cameo, Silhouette, Brazil) was used to create the electrode pattern drawn via Silhouette Studio v. 4.3 software. The ePAD electrode morphology and structure were investigated by scanning electron microscopy (SEM, Quanta 400 and FE-SEM, Apreo, FEI, USA) operating at 20 and 50 kV. A Fourier-transform infrared (FTIR) spectrometer (VERTEX 70, Bruker, Germany) was used with a KBr pellet, and absorbance was captured at wavenumbers between 400 and 4000 cm^–1^ at a resolution of 4 cm^–1^. All electrochemical determinations were performed with the lab-built portable device for xylazine analysis (Scheme 1).

### ePAD Fabrication Process

**Figure 1:**
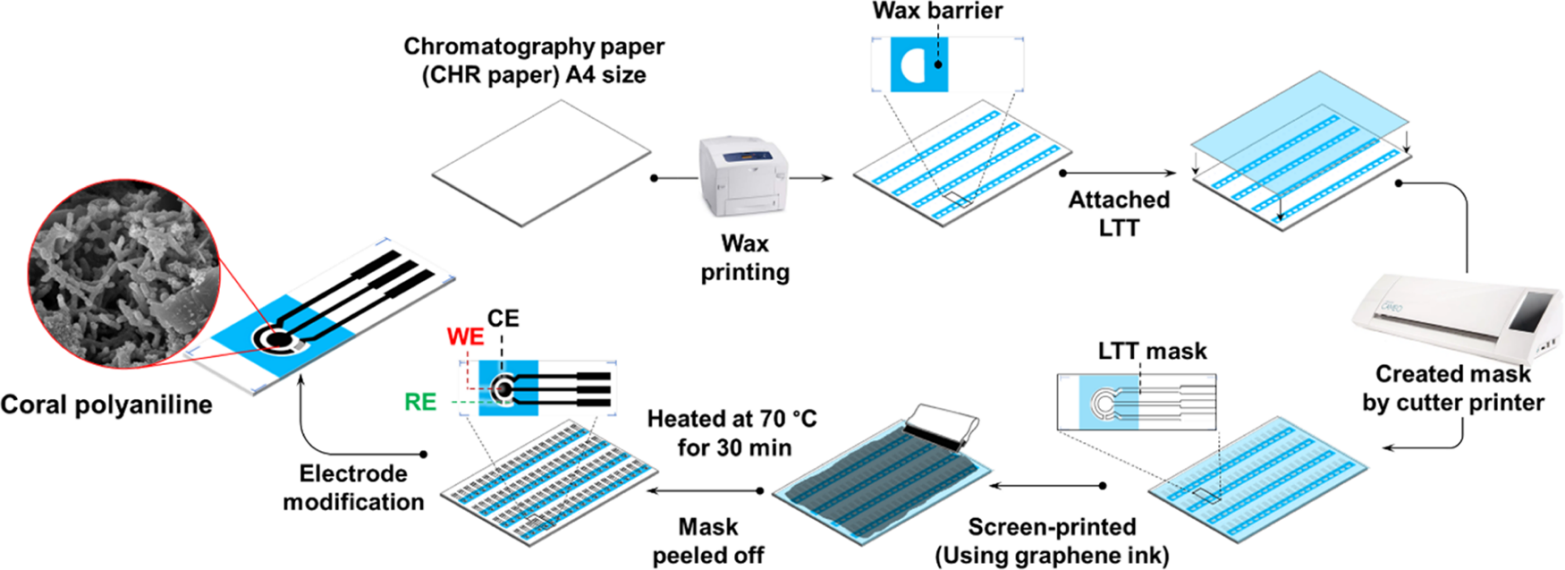
Schematic representation of the ePAD fabrication process.

The ePAD was fabricated following a previously reported method^22^ (Figure 1). In brief, rows of hydrophobic barriers were created by printing wax on a CHR paper that was then heated with a hot air dryer. LTT was placed on top of the CHR paper to cover the hydrophobic barriers, and using a printer cutter, negative masks of the ePAD electrode [three-electrode system consisting of a working electrode (WE), a pseudo-reference electrode (RE), and a counter electrode (CE)] were cut out of the LTT to expose the CHR paper. The LTT was then coated with graphene ink using a squeegee. The ink was forced through the mask onto the CHR paper, creating a series of graphene ink electrode patterns 1.25 cm wide and 3.75 cm long. After curing the ink for 30 min at 70 °C, Ag/AgCl ink was applied with a paintbrush to the RE areas to create the RE on each electrode pattern. The paper was then heated in an oven at 70 °C for 30 min. The LTT mask was carefully peeled off, and the electrode patterns were cut out of the CHR paper. Finally, the individual electrode patterns were modified with coral-like PANI to create a modified ePAD ready for use in a portable electrochemical sensor (Scheme 1) for on-site xylazine detection and analysis.

The portable electrochemical sensor used in this work was adapted from our work.^22,31^ The sensor housing was designed in a pill bottle box case (Scheme 1) to accommodate the disposable ePAD sensor and a USB connector that can plug into a mobile phone loaded with the appropriate software application. The developed device was made up of three parts (Figure S1). The sensing device was equipped with an Emstat Pico Module potentiostat to provide the potential to the PANI/ePAD sensor and to measure the generated current. The xylazine sensor software application, developed from Software Development Kits (SDKs) for.NET (www.palmsens.com/oem/sdkdotnet/), was installed on the portable monitoring device and controlled the operation of the sensing device. The third part was the PANI-modified ePAD sensor that detected the presence of xylazine and generated the electrochemical signal.

### Synthesis of Coral-like PANI

A coral-like PANI composite was synthesized by the polymerization of the aniline monomer in the presence of a 25% NaCl solution. To 100 mL of the NaCl solution were added 18 mL of concentrated hydrochloric acid (HCl) and 1.82 mL of aniline monomer. The precipitate of NaCl was then dissolved in a few drops of DI water. In the next step, 4.56 g of ammonium persulfate (APS) was added dropwise to 100 mL of the NaCl solution for about 15 min, and the mixture was stirred for 12 h. The product was filtered, washed first with 500 mL of DI water and then with 250 mL of ethanol, and dried in an oven at 60 °C for 12 h. Finally, coral-like PANI was suspended in DMF to a concentration of 2.0 mg mL^–1^, dropped onto the WE area of the ePAD, and allowed to dry at 70 °C for 5 min.

### Electrochemical Measurements

The electrochemical measurements were performed by dropping 30 μL of BR buffer (pH 7.00) containing various concentrations of xylazine covering three electrodes in the detection zone of the ePAD. Cyclic voltammetry (CV) was carried out by scanning a potential from +0.30 to +1.00 V at a scan rate of 0.05 V s^–1^. The analysis of xylazine in beverage samples was performed using differential pulse voltammetry (DPV) under the following conditions: E pulse 0.20 V, t pulse 250 ms, E step 0.02 V, and scanning between +0.20 V and + 0.90 V at a scan rate of 0.03 V s^–1^. Electrochemical impedance spectroscopy (EIS) was also performed with a frequency range from 0.05 to 50,000 Hz, a frequency number of 50, an *E*_dc_ of +0.25 V, and an *E*_ac_ of +0.01 V.

### Sample Analysis

Xylazine was spiked at 5, 10, 20, 30, and 40 μg mL^–1^ into separate beverage samples that comprised non-alcoholic and alcoholic products available in supermarkets. The products included Calpis Lacto (pH 4.76), OISHI (pH 6.67), Pepsi Max (pH 3.35), Yanhee Vitamin water (pH 7.25), Soda Rock Mountain (pH 7.24), Smirnoff Gold (4% alcohol, pH 3.43), and Jinro Chamisul Soju (17% alcohol, pH 7.85). A 2 mL aliquot of the spiked sample was added to 2.0 mL of BR buffer at pH 7.00 and manually shaken. A 30 μL aliquot was transferred onto the detection zone of the ePAD, and the detection and quantification of xylazine were carried out via the portable electrochemical sensor.

## Results and Discussion

### ePAD Fabrication and Characterization

**Figure 2:**
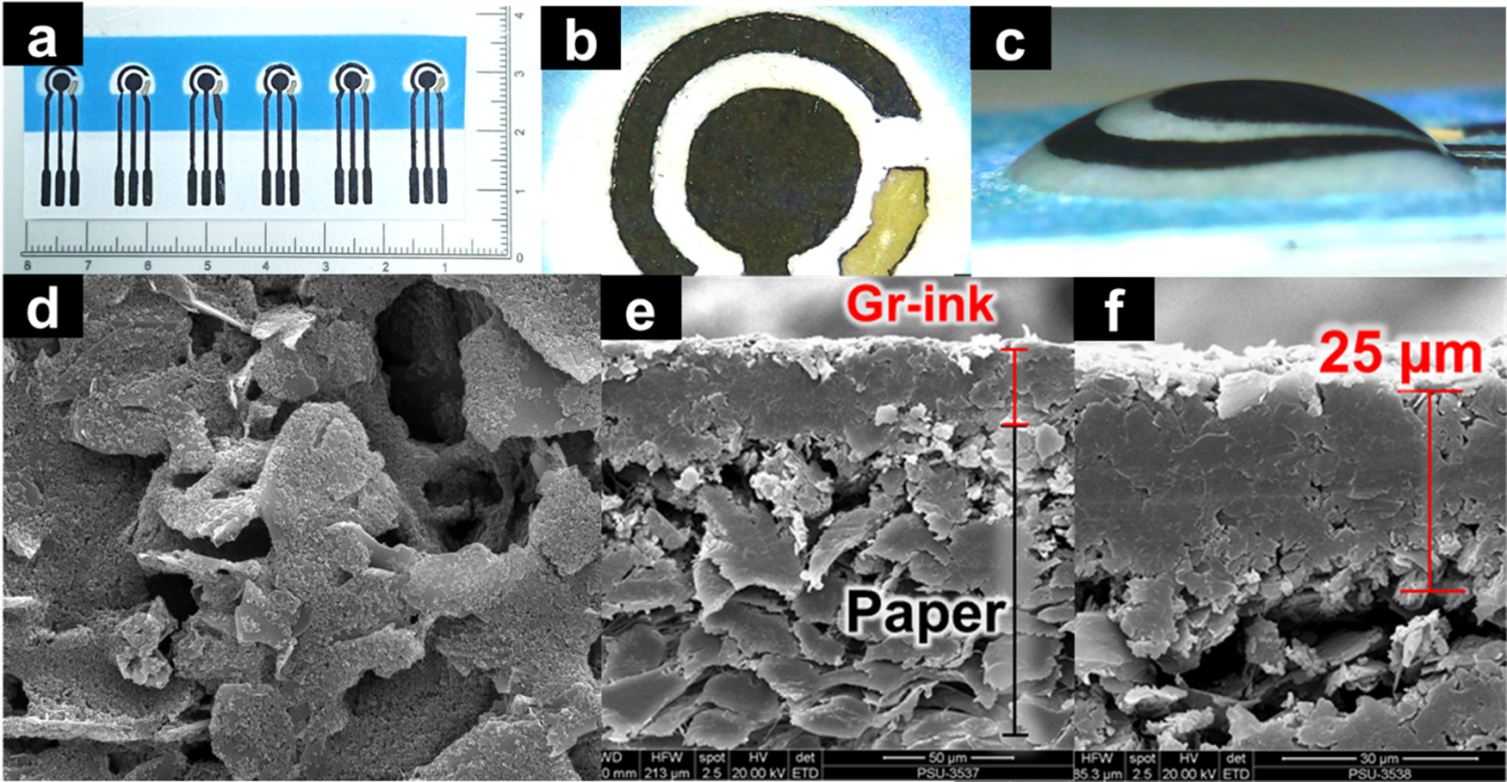
The digital images (a–c) show six graphene ink-based three-electrode devices fabricated on a chromatography paper; the WE, RE, and AE of a device; and the action of the wax barrier in the presence of water. The SEM images show the surface of a graphene ink WE (d) and cross sections (e,f) of the WE of a fabricated ePAD.

The ePAD was fabricated by a simple procedure using an inexpensive craft printer/cutter and LTT to create a mask template for the screen-printing process. Figure 2 shows digital and SEM images of the ePAD. The three graphene ink electrodes of the ePAD showed a well-defined geometry (the individual ePADs, which are approximately 1.25 cm wide and 3.75 cm long), indicating the suitability of LTT as a template mask. Figure 2a shows a digital image of the fabricated ePAD. The successful construction of simple and flexible electrodes can be seen. The WE (diameter = 3 mm; geometric surface area = 0.071 cm^2^), RE (diameter = 0.75 mm; geometric surface area = 0.015 cm^2^), and AE (diameter = 0.75 mm; geometric surface area = 0.058 cm^2^) were well defined, as shown in Figure 2b, where the blue region is the wax barrier. In Figure 2c, the detection zone can be seen completely filled with water inside the wax barrier. Figure 2d displays an SEM image showing the morphology of the screen-printed graphene ink WE on an ePAD. The rough surface provided a large, active, and electrically conductive surface area for electrochemical analysis. The cross-sectional image in Figure 2e reveals the thickness of the graphene ink layer compared to the thickness of the CHR paper. Figure 2f shows the average thickness of the graphene ink layer, measured at 25.0 ± 0.9 μm.

### Nanocoral-like PANI/ePAD Morphology and Electrochemical Characterization

**Figure 3:**
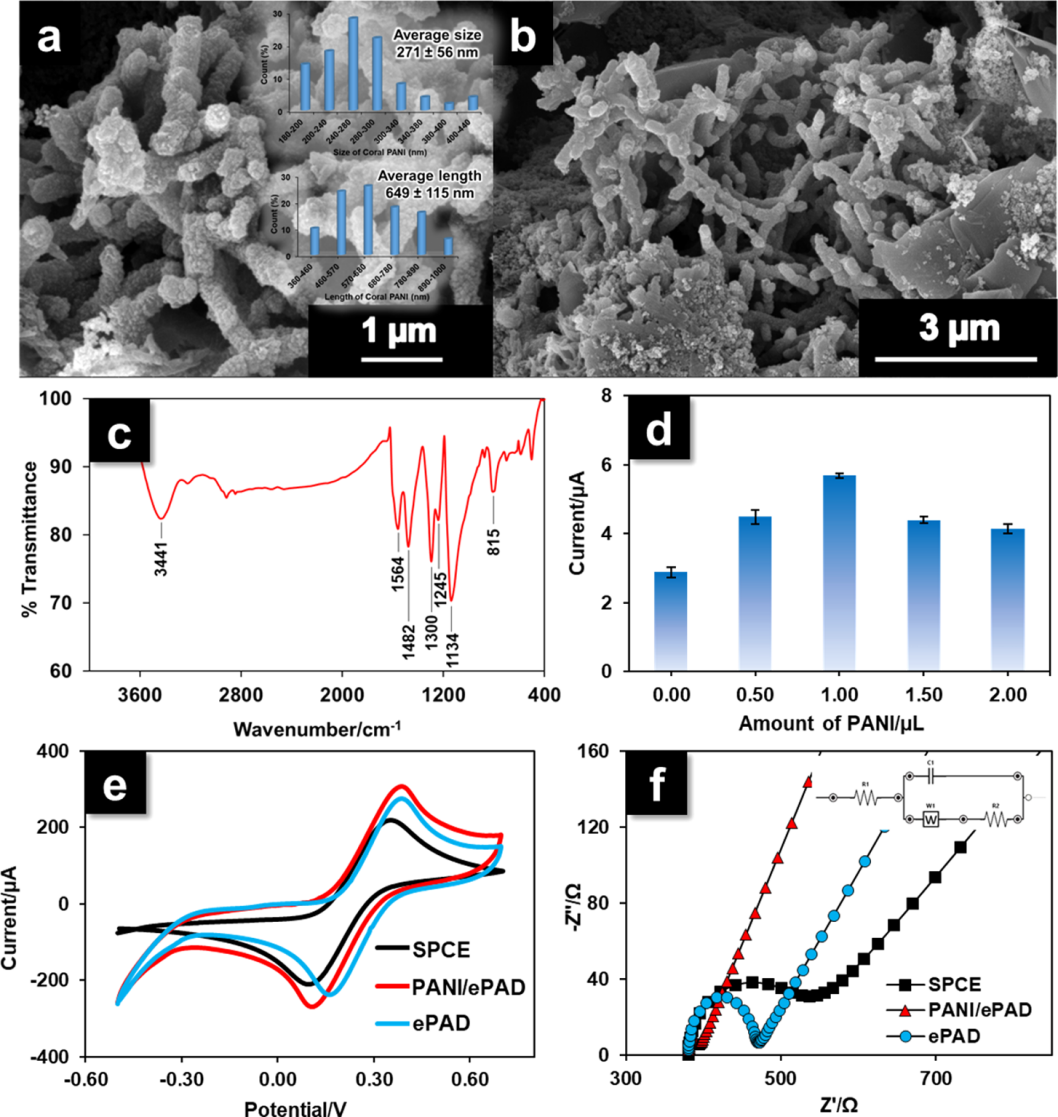
FE-SEM images are of (a) coral-like PANI (inset histograms show the diameter and length distributions of coral-like PANI) and (b) ePAD surface modified with coral-like PANI. The FTIR spectrum (c) is of coral-like PANI. The histogram (d) shows the effect of PANI loading on the ePAD (0.00–2.00 μL) (*n* = 3). CV (e) and EIS (f) results were produced at an SPCE, a bare ePAD, and a PANI/ePAD in 0.10 M KCl containing 10 mM [Fe(CN)_6_]^3–/4–^ [inset section is an equivalent circuit (Randle circuit) used for fitting the EIS spectra].

The surface morphology of the coral-like PANI was characterized using FE-SEM. Figure 3a shows a coral-like structure with a highly porous and interconnected network produced by the polymerization of PANI in the NaCl solution. The average diameter and length of the coral-like PANI structures (inset Figure 3a), measured with an electronic digital caliper on an enlarged FE-SEM micrograph, were 271 ± 56 and 649 ± 115 nm, respectively. Drop-casting PANI onto the WE surface of the ePAD successfully incorporated the coral-like structure into the rough surface of the graphene ink, as shown in Figure 3b. Figure 3c displays the FTIR spectrum of PANI. The peaks at 1564 and 1482 cm^–1^ were attributed to the C=C stretching of quinoid and benzenoid rings, respectively. The peaks at 1300 and 1245 cm^–1^ were produced by C–N stretching vibrations, and the peaks at 1143 and 815 cm^–1^ were, respectively, due to C–C stretching and C–H out-of-plane bending in the chemical structure.

Because the amount of coral PANI on the electrode surface could influence the adsorption capacity, sensitivity, and the limit of detection (LOD) of the sensor, the PANI loading was optimized by measuring the electrochemical signal toward xylazine at electrodes loaded with 0.0, 0.5. 1.0, 1.5, and 2.0 μL of PANI. The current signal increased with coral PANI loading from 0.0 to 1.0 μL and decreased at higher loadings (Figure 3d). An increase in the volume of PANI resulted in more adsorption sites. The adsorption of xylazine on PANI mainly occurred on the amino groups of the chemical structure by hydrogen bonding and on the benzene ring by π–π stacking.^32^ Higher loadings resulted in lower current generation, probably because the increased thickness of the modified electrode inhibited the electron transfer. Therefore, the optimum drop-cast solution volume of PANI was determined at 1.0 μL.

Additionally, the electrochemical properties of the PANI-modified ePAD were studied using CV to compare the electrochemical activities of an SPCE and a bare ePAD and a PANI/ePAD in 0.1 M KCl containing 10 mM [Fe(CN)_6_]^3–/4–^. As shown in Figure 3e, the redox peak current of PANI-modified ePAD (red line) showed significantly higher redox peak currents (*I*_p_ = 303 μA) than the bare ePAD (*I*_p_ = 269 μA), indicating that the PANI modified on the ePAD significantly increases the electrochemical sensitivity of the system.^33^ Interestingly, the peak-to peak potential separation (ΔE_p_) of [Fe(CN)_6_]^3–/4–^ on the ePAD (Δ*E*_p_ = 221 mV; blue line) significantly decreases when compared to Δ*E*_p_ obtained from commercial SPCE (Δ*E*_p_ = 254 mV), indicating good electrical conductivity and relatively larger surface area of the graphene ink on the ePAD.

The active surface areas of the bare SPCE (geometric surface area = 0.125 cm^2^; *d* = 4 mm), ePAD (geometric surface area = 0.071 cm^2^; *d* = 3 mm), and PANI/ePAD (geometric surface area = 0.071 cm^2^; *d* = 3 mm) were calculated from the slope of the plot of *I*_pa_ versus *v*^1/2^ and the Randles–Sevcik equation, *I*_p_ = (2.69 × 10^5^) *An*^3/2^*D*^1/2^*C*υ^1/2^, where *n* is the electron transfer number, *A* is the surface area of the electrode, *D* is the diffusion coefficient, *C* is the concentration of [Fe(CN)_6_]^3–/4–^, and υ is the scan rate. For 10 mM [Fe(CN)_6_]^3–/4–^, *n* was determined to be 1, and the *D* value for [Fe(CN)_6_]^4–^ was 6.67 × 10^–6^ cm^2^ s^–1^,^34^ based on the plot of anodic peak current versus the square root of scan rate. Therefore, the active surface areas of the bare SPCE, bare ePAD, and PANI/ePAD were, respectively, 0.321, 0.352, and 0.390 cm^2^. These results confirmed that the PANI/ePAD had a larger effective surface than the other electrodes and should perform well in xylazine sensing.

EIS is an effective technique to monitor the electrochemical properties of electrode surfaces. Figure 3f displays the impedance plots (Nyquist plots) of an SPCE, a bare ePAD, and a PANI/ePAD recorded in 0.10 M KCl containing 10 mM [Fe(CN)_6_]^3–/4–^. The obtained Nyquist plots (imaginary impedance -*Z*″ vs real impedance Z′) were analyzed by the Randles equivalent circuit, as shown in the inset of Figure 3f. The equivalent circuit compatible with the EIS data consists of *R*_S_, *R*_CT_, *W*, and CPE_dl_, symbolizing the resistivity of the solution, charge transfer resistance, Warburg impedance, and constant phase element corresponding to the capacitance of the electric double layer, respectively. Using such an equivalent circuit, *R*_CT_ values are determined. In the case of ePAD, the *R*_CT_ value is 84.9 Ω, indicating a lower resistance than SPCE (*R*_CT_ = 160.6 Ω), which means that it also displayed the high electrical conductivity of ePAD. Moreover, after the modification ePAD with PANI, the diameter of the semicircle is found to decrease by exhibiting a massive reduction in the R_CT_ value of 11.6 KΩ. The results confirmed the considerably higher conductivity of the PANI/ePAD, where electron transfer at the electrode had been improved.

### Electrochemical Oxidation of Xylazine at PANI/ePAD

**Figure 4:**
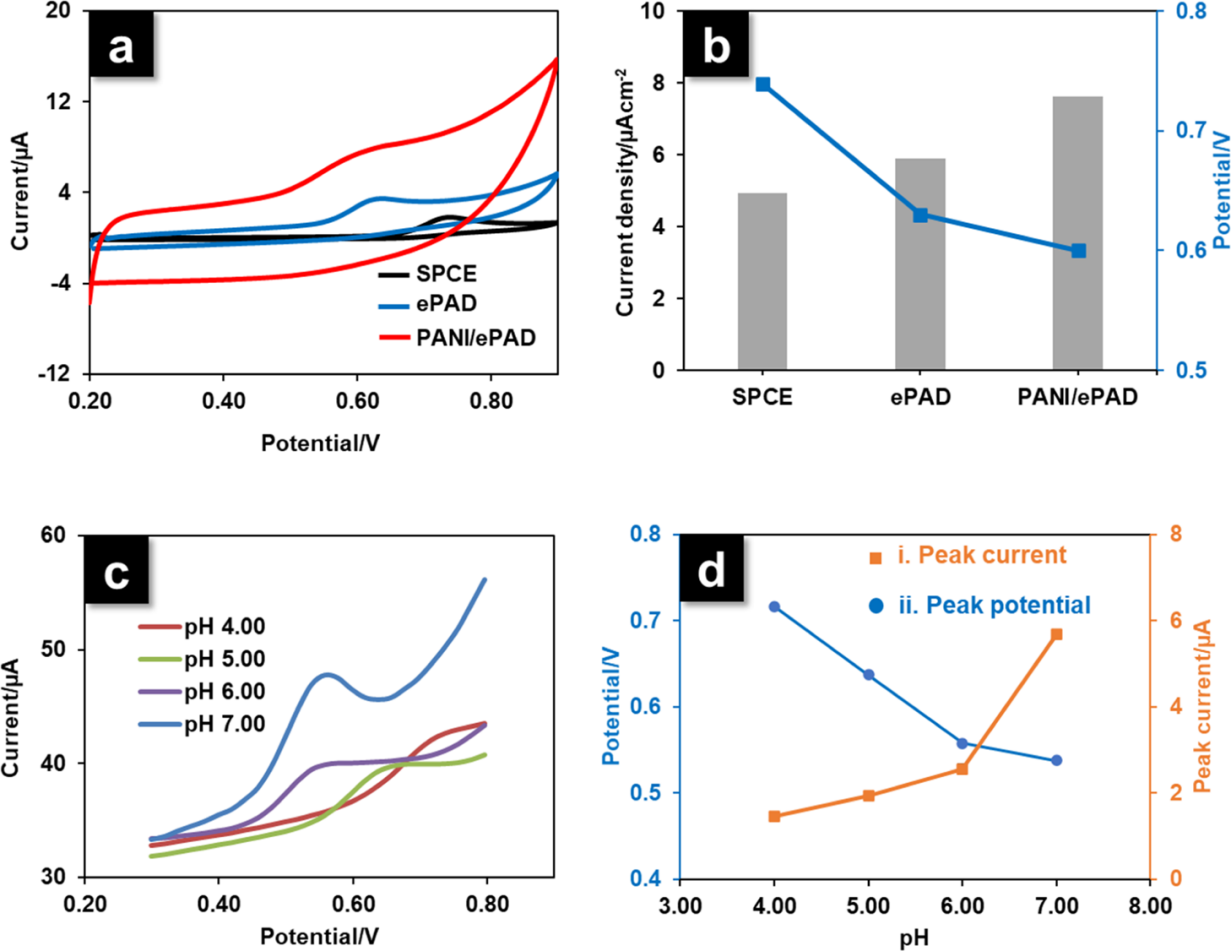
(a) CVs were obtained from an SPCE, a bare ePAD, and a PANI/ePAD in BR buffer at pH 7.00 containing 10 μg mL^–1^ xylazine. (b) Comparison of the current density and the peak potential produced at the SPCE, bare ePAD, and PANI/ePAD. (c) Differential pulse voltammograms of xylazine (10 μg mL^–1^) were produced at the PANI/ePAD in BR buffer at different pH values (4.00–7.00). (d) Relationship between *I*_pa_ (i) and *E*_pa_ (ii) vs pH (*n* = 3).

The electrochemical behavior of xylazine was evaluated at SPCE, bare ePAD, and PANI/ePAD. CV was applied using potentials from +0.20 to +0.90 V at a scan rate of 0.05 V s^–1^. The voltammograms of 10 μg mL^–1^ xylazine produced at all three electrodes indicated that the oxidation of xylazine was an irreversible electrode reaction mechanism (Figure 4a). The peak potential of xylazine at SPCE was +0.74 V, at bare ePAD was +0.64 V, and at PANI/ePAD was +0.60 V, as presented in Figure 4b. A significant increase in the peak current of xylazine was correlated with the surface area and conductivity of the electrodes. In addition, with regard to xylazine oxidation, PANI/ePAD produced a greater anodic peak current than the bare ePAD and SPCE, showing that modification with PANI augmented the electrode function toward the oxidation of xylazine.

### Effect of pH

The electrochemical behavior of xylazine in BR buffer at different pH values is shown in Figure 4c. The influence of pH on the current response of xylazine in BR buffer at pH 4.00–7.00 was established using DPV at the PANI/ePAD (Figure 4di). The peak current of xylazine decreased with reductions in pH from 7.00 to 4.00, which was presumably due to the partial protonation of secondary amines in the xylazine structure at a lower pH (p*K*a of xylazine: 6.94). In contrast, at pH higher than 7.00, the solution tended to turn turbid, which was perhaps related to the hydrolysis or degradation of the compound.^19^ Thus, we chose BR buffer at pH 7.00 for xylazine electro-oxidation at the PANI/ePAD surface. The change in the anodic peak potential (*E*_p_) for the oxidation of xylazine as a function of pH is presented in Figure 4dii. A negative shift was observed in the oxidation peak potential with the increase in pH, which suggests that protons participate in the electrode reaction process.

### Kinetic Mechanism of Xylazine on PANI/ePAD

**Figure 5:**
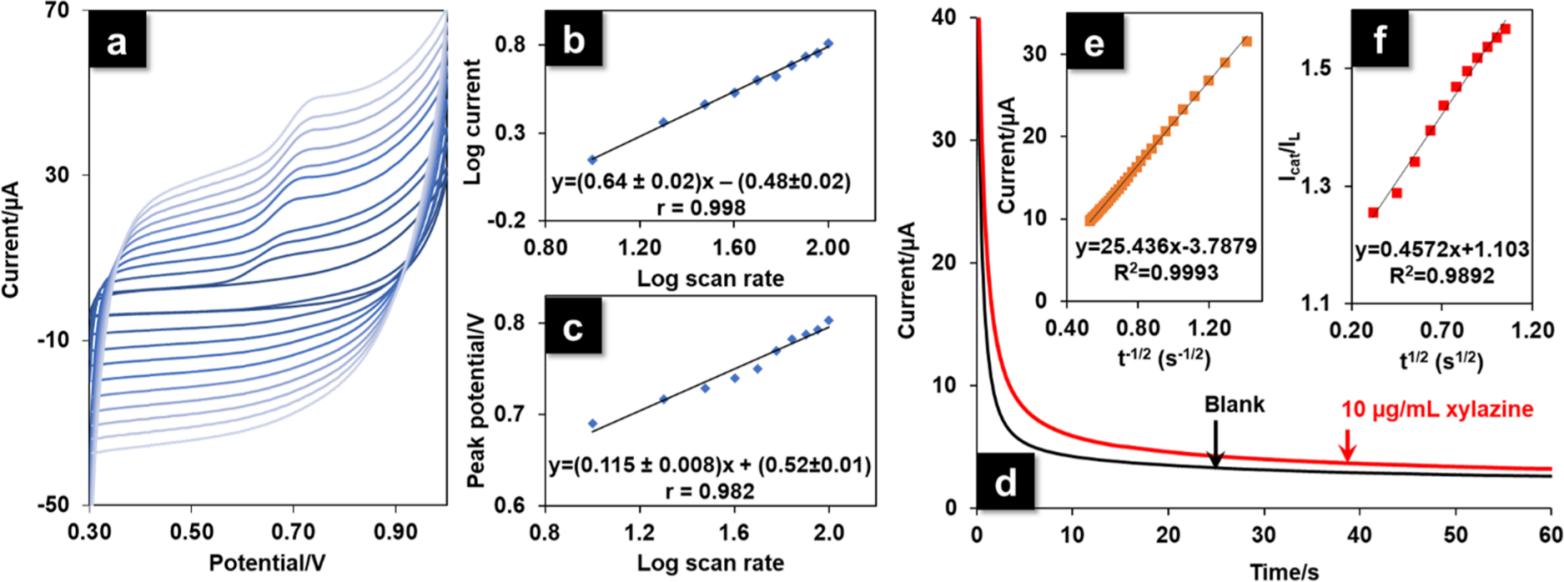
(a) CV curves were produced at scan rates from 20 to 200 mV s^–1^ at the PANI/ePAD in BR buffer at pH 7.00 containing 10 μg mL^–1^ xylazine. (b) Plot of log *I* vs log υ. (c) Plot of peak potential vs log υ. (d) *i*–*t* curves of the PANI/ePAD with and without 10 μg mL^–1^ xylazine at 0.70 V. (e) Plot of *I* vs *t*^–1/2^ and (f) plot of *I*_cat_/*I*_L_ vs *t*^1/2^.

We applied CV at scan rates from 20 to 200 mV s^–1^ to evaluate the electrochemical kinetic behavior of xylazine on the PANI/ePAD by studying the influence of the scan rate on the peak current and peak potential for 10 μg mL^–1^ xylazine in BR buffer at pH 7.00 (Figure 5a). The relationship between the log peak current and log scan rate (log *I*_p_ vs log υ) was used to evaluate the kinetic behavior of xylazine at the PANI/ePAD interface. The linear relationship of log *I*_p_ versus log υ, shown in Figure 5b, was log *I*_p_ = (0.64 ± 0.02) log υ – (0.48 ± 0.02); *r* = 0.998. The obtained slope value was between 0.5 (purely a diffusion-controlled process) and 1.0 (purely an adsorption-controlled process). This result indicated a combination of diffusive and adsorptive behaviors. The good linearity of both *I*_p_ versus υ (adsorption-controlled process) and *I*_p_ versus υ^1/2^ (diffusion-controlled process), shown in Figure S2a,b, corresponds to the results obtained from the Randles–Sevcik equation. The slight difference between the linear relationship of *I*_p_ versus υ^1/2^ (*r* = 0.993) and the linear relation of *I*_p_ versus υ (*r* = 0.995) was probably due to the combination of diffusion and adsorption processes that typified the electrochemical behavior of xylazine upon oxidation at the surface of the PANI/ePAD.

From the Tafel slopes of the totally irreversible process (Figure S2c), the value of *b* for the PANI/ePAD was 0.427 V dec^–1^. The Tafel value at the PANI/ePAD was higher than the theoretical value of 0.118 V dec^–1^ for a one-electron process involved in the rate-determining step.^35^ Therefore, the high Tafel value suggested the adsorption of xylazine or its reaction intermediate at the electrode surface. In the literature, high Tafel values have been attributed to the adsorption of reactants or intermediates on electrode surfaces and/or reactions within an electrode structure.^26,35^

In addition, from the linear relationship of *I*_p_ versus υ, the slope could be used to estimate the surface concentration of electroactive species (Γ) by using eq 1.^36^ The value of Γ on the surface of the PANI/ePAD was found to be 2.04 × 10^–7^ mol cm^–2^.
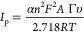
1

The number of electrons involved in the oxidation reaction of xylazine on the surface of the PANI/ePAD was calculated from Laviron’s eq 2,^37^ based on the slope of the plot of *E*_p_ versus log υ, as shown in Figure 5c.
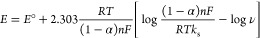
2where *F*, *R*, *T*, α, and *n* are the Faraday constant, the gas constant, the temperature, the charge transfer coefficient, and the number of electrons, respectively. The slope value of the oxidation peak was 0.115 (Figure 5c). Thus, the *n* value was calculated to be ≈1, indicating that one electron was involved in the oxidation of xylazine on the PANI/ePAD. This result was in agreement with previous reports.^20,21^

To evaluate the diffusion coefficient of xylazine at the PANI/ePAD, xylazine at 10 μg mL^–1^ was measured in BR buffer at pH 7.00 by chronoamperometry at +700 mV. Cottrell’s eq 3 was applied to calculate the diffusion coefficient (*D*)^35^
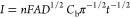
3where *D* is the diffusion coefficient of the analyte (cm^2^ s^–1^), *C*_b_ is the analyte bulk concentration (mol cm^–3^), *F* is the Faraday constant, *n* is the number of electrons, and *A* is the electrode geometric area. Plotted from the raw chronoamperometric data (Figure 5d), Figure 5e shows the linear curves of *I* versus *t*^–1/2^. The diffusion coefficient of xylazine on the PANI/ePAD was calculated to be 7.74 × 10^–6^ cm^2^ s^–1^.

The electrocatalytic performance of the PANI/ePAD for the electrochemical oxidation of xylazine was evaluated from the catalytic rate constant (*k*_cat_), calculated using Galus’s eq 4(38)
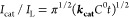
4where *I*_cat_ is the catalytic current for xylazine, *I*_L_ is the limited current in the absence of xylazine, *t* is the time elapsed, and *C*^0^ is the bulk concentration of xylazine. From the slope of *I*_cat_/*I*_L_ versus *t*^1/2^ (Figure 5f), the ***k***_**cat**_ value of the PANI/ePAD for xylazine oxidation was determined to be 1.48 × 10^5^ M^–1^ s^–1^. The relatively high values for the diffusion coefficient and catalytic rate constant, which indicated greater electrocatalytic efficiency for xylazine detection, could be attributed to the coral structure of PANI on the porous graphene ink of the ePAD. Thus, the use of the PANI/ePAD in the developed electrochemical sensor had enhanced sensitivity toward xylazine.

### Optimization of the Electrochemical Parameters

Electrochemical parameters of the developed xylazine sensor were optimized to improve the performance and efficiency of the system. Optimizations were carried out by changing one parameter while keeping the other parameters constant. Parameters tested included the DPV conditions, namely, the pulse potential, pulse time, applied scan rate and step potential, and the accumulation step covering both the potential and the time. The highest current signal obtained from the measurement of 10 μg mL^–1^ xylazine at each setting was considered to indicate the optimal condition.

### Effect of Differential Pulse Parameters

In this study, DPV was applied for its intrinsic high current sensitivity and low charging toward the formation of background current. Pulse time (*t*_pulse_), pulse potential (*E*_pulse_), step potential (*E*_step_), and applied scan rate were investigated with the aim of increasing the current response.

**Figure 6:**
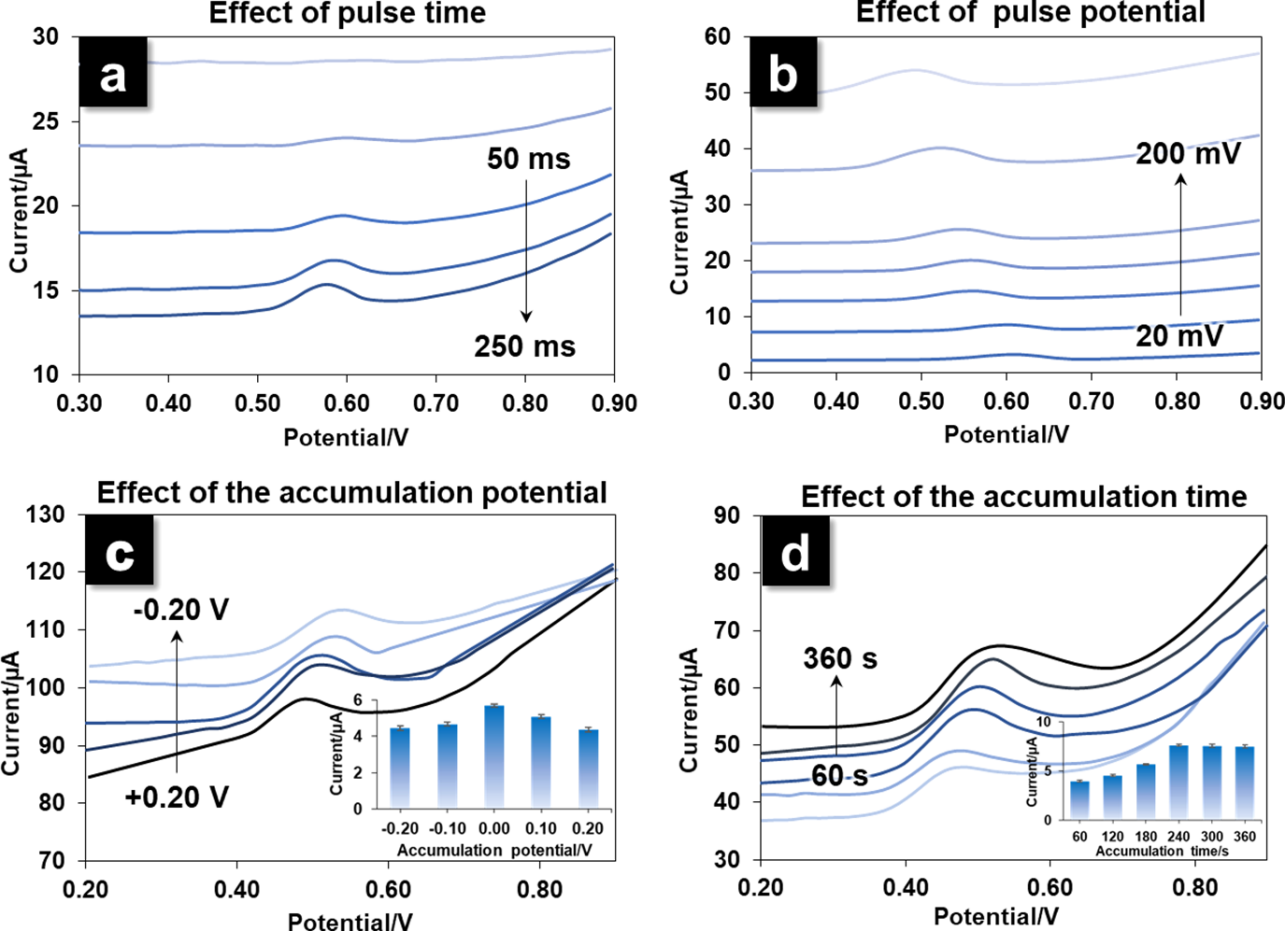
Voltammograms from DPV show the effect of pulse time (a), pulse potential (b), accumulation potential (c), and accumulation time (d) on the current response of 10 μg mL^–1^ xylazine at the PANI/ePAD in BR buffer at pH 7.00 (*n* = 3 for each experiment).

The effect of *t*_pulse_ (50–250 ms) was studied by measuring the current response of 10 μg mL^–1^ of xylazine at the PANI/ePAD using a constant *E*_pulse_ (40 mV), *E*_step_ (20 mV s^−1^), and applied scan rate (40 mV s^−1^), as shown in Figure 6a. The current signal was observed starting at *t*_pulse_ of 100 ms and increased from 100 to 250 ms. At longer pulse times, the background current was found to decrease, forming a sharper anodic current peak. At a *t*_pulse_ of 250 ms, the effect of *E*_pulse_ was evaluated in the range from 20 to 200 mV (Figure 6b).

The current signal was found to increase with increments in the pulse potential within the tested range. However, the peak width was also found to increase along with a negative shift in the anodic peak potential. Evaluation of the effect of *E*_step_ (20–40 mV) and applied scan rate (10–50 mV s^−1^) showed that the analysis time was shorter with a larger step potential and scan rate, but the decaying charge current was also low, causing higher background current in the DPV. Therefore, an *E*_step_ of 20 mV, an applied scan rate of 30 mV s^−1^, a *t*_pulse_ of 250 ms, and an *E*_pulse_ of 200 mV were the parameters used in the next experiment.

### Effect of Accumulation Potential and Accumulation Time

The sensitivity and LOD of xylazine were greatly improved by using adsorptive stripping voltammetry (AdSV). The effects of the accumulation step, comprising accumulation potential and time, were investigated. The accumulation potential was investigated between −0.20 and +0.20 V over an accumulation time of 180 s. Figure 6c shows the voltammograms of xylazine oxidation at different accumulation potentials, where the background current increased when the accumulation potential was increased to a negative potential (+0.20 to −0.20 V). This behavior could be caused by the increase in the charging current on the electrode surface when the accumulation potential was more negative. It also led to a significant increase in the background current, mainly due to the catalytic decomposition of the electrolyte.^39^ The highest current was recorded at 0.00 V (vs Ag/AgCl) (inset Figure 6c), and this potential was used in the subsequent analysis of accumulation time from 60 to 360 s. Figure 6d shows the voltammograms of xylazine oxidation at different accumulation times, where a continuous increase in the xylazine current with the accumulation time was evident from 60 to 240 s and no significant change was observed beyond 240 s (inset Figure 6d). This result was probably due to the saturation of xylazine on the PANI/ePAD surface. Another explanation could be the increase in the background current that occurred with the increase in the accumulation time. At extended accumulation times, adsorption on the electrode surface was no longer limited to xylazine but also included the charging ion, producing a large increase in the background current and making the determination of xylazine by electro-oxidation more difficult.^39^ Therefore, an accumulation potential of 0.00 V and an accumulation time of 240 s were chosen as the optimal conditions.

### Analytical Performances

**Figure 7:**
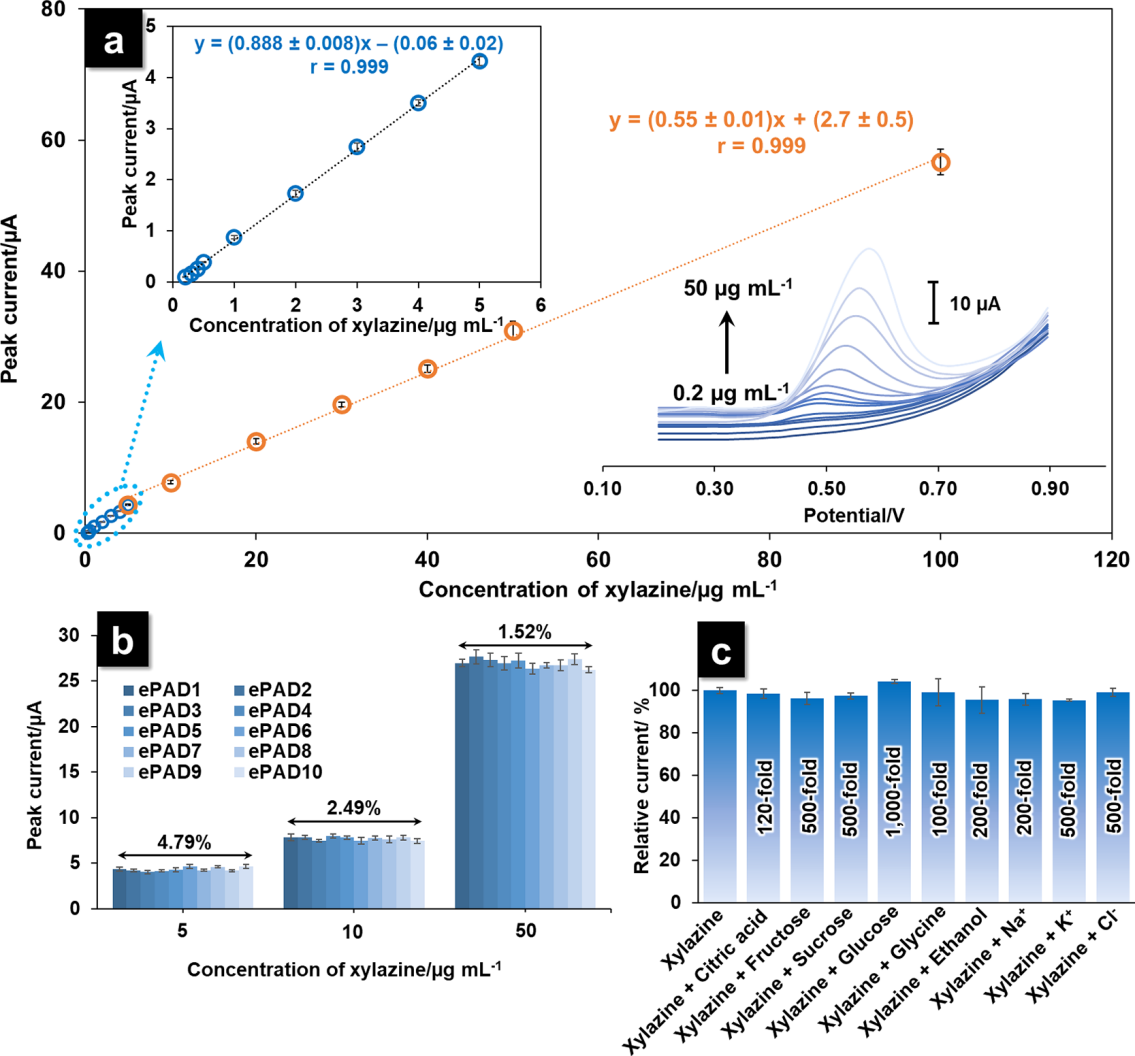
The calibration curve (a) is of the current response of xylazine at concentrations from 0.2 to 100 μg mL^–1^ (*n* = 3): inset shows the calibration plot of the lower linear range from 0.2 to 5 μg mL^–1^. (b) Relative current response of 10 PANI/ePAD preparations (*n* = 3 for each electrode). (c) Influence of possible interfering species on the peak current of 10 μg mL^–1^ xylazine (*n* = 3 for each interfering species).

Analytical performances of the PANI/ePAD for xylazine detection were investigated using AdSV based on the optimized conditions. Figure 7a shows the anodic peak current of xylazine at concentrations from 0.2 to 100 μg mL^–1^. The anodic peak current of xylazine was observed at a potential of +0.52 V. The current increased linearly with increments in xylazine concentration, and two linear ranges of xylazine detection were presented at concentration ranges of 0.2–5 and 5–100 μg mL^–1^. The occurrence of two linear ranges was due to the adsorption behavior of xylazine. At lower concentrations, the target substance was adsorbed as a monolayer on the PANI/ePAD surface, and at higher concentrations, xylazine was adsorbed as a double layer or multilayer.^40^ The LOD and limit of quantitation (LOQ) of the developed method were calculated from the equation LOD = 3 (S.D_blank_/slope) and LOQ = 10 (S.D_blank_/slope), respectively. Here, the LOD and LOQ were found to be 0.06 and 0.21 μg mL^–1^, respectively. In comparison to other techniques for determining xylazine reported in the literature (Table 1), our PANI/ePAD provided a wide linearity and low LOD. In addition, the developed sensor was simpler to fabricate and use, easily portable and can be used forensically to determine xylazine in beverages.

**Table 1:** Comparison of the Analytical Performances of Previously Reported Methods for the Detection of Xylazine

technique	detection potential (V)	linear range (μg mL^–1^)	LOD (μg mL^–1^)	sensitivity	reproducibility (% RSD)	sample	application	on-site analysis	refs
aPANI/ePAD with DPV	+0.52	0.2–5.0 and 5.0–100.0	0.06	12.51 μA μg^–1^ mL cm^–2^	1.52–4.79	beverage	forensic	yes	this work
bGNPs/SPCE with LSV	+0.73	0.4–6.0 and 6.0–80.0	0.10	10.42 μA μg^–1^ mL cm^–2^	3.57–6.85	beverage	forensic	yes	(21)
cGCE with DPV	+0.85	0.1–56.0	0.03	0.38 μA μg^–1^ mL cm^–2^	3.8	urine	forensic	no	(19)
dMWCNT-BMH-SDS/CPE with DPV	+1.00	0.01–0.32	0.001	2.62 μA ng^–1^ mL^–1^		human blood	pharmaceutical	no	(20)
eHPLC-UV		0.01–5.00	0.02		1.9–2.0	canine plasma	veterinary	no	(17)
fGC–MS		0.05–1.50	0.04			equine urine	veterinary	no	(6)
gLC/MS/MS		5 × 10^−5^–1× 10^-1^	6 × 10^–5^		<10	animal tissues	veterinary	no	(11)

a PANI/ePAD with DPV: polyaniline-modified electrochemical paper-based analytical device with differential pulse voltammetry.

b GNPs/SPCE with LSV: graphene nanoplatelet-modified screen-printed carbon electrode with linear sweep voltammetry.

c GCE with DPV: glassy carbon electrode with differential pulse voltammetry.

d MWCNT-BMH-SDS/CPE with DPV: multiwall carbon nanotube/1-*n*-butyl-3-methylpyridinium hexafluorophosphate ion crystal/sodium dodecyl sulfate on carbon paste electrode with differential pulse voltammetry.

e HPLC-UV: high-performance liquid chromatography with ultraviolet absorbance detection.

f GC–MS: gas chromatography coupled with mass spectrometry.

g LC/MS/MS: liquid chromatography–tandem mass spectrometry.

The reproducibility of the PANI/ePAD was assessed through the evaluation of 10 electrode preparations. When comparing the peak current from 10 repetitions of the electrode, good reproducibility was reported with relative standard deviations (RSD) from 1.52 to 4.79% (Figure 7b). The reported RSDs were within an acceptable range according to the guidelines of the Association of Analytical Communities (AOAC).^41^

The effects of interferences on xylazine determination with the developed electrochemical sensor were evaluated by measuring various interfering compounds that might be present in beverage samples (citric acid, fructose, sucrose, glucose, glycine, ethanol, Na^+^, K^+^, and Cl^–^) in the presence of 10 μg mL^–1^ xylazine (Figure S3a). The results (Figure 7c) showed no interference in the presence of 1200-fold of citric acid, 1000-fold of glucose, 100-fold of glycine, 200-fold of ethanol and Na^+^, and 500-fold of fructose, sucrose, K^+^, and Cl^–^. The results of this study indicated the good anti-interference property of the proposed device. Furthermore, the selectivity of the PANI/ePAD was investigated under the optimal conditions by comparing it to other similar compounds such as benzodiazepine class (i.e., alprazolam, diazepam, and clonazepam), pseudoephedrine, and methamphetamine. Figure S3b shows that there is no significant current signal using the PANI/ePAD sensor. This finding indicates that the PANI/ePAD electrode is highly selective for xylazine.

### Xylazine Detection in Samples

The practicability of the proposed portable sensor was demonstrated by measuring the levels of xylazine in selected alcoholic and non-alcoholic beverage samples spiked with standard xylazine. The matrix effect of each beverage sample was studied in the optimized condition by comparing the slope of a standard curve of each beverage compared with a standard xylazine calibration curve. The data were analyzed by a two-way ANOVA. The results showed no significant difference at a confidence level of 95%, which indicated that there was no matrix effect. Consequently, the amount of xylazine in each beverage sample could be deduced using the linear regression equation of the standard curve, and the percentage recovery values were then calculated. The recovery values among all the tested samples ranged from 84 ± 4 to 105 ± 2% (*n* = 3), as shown in Table 2. The good recovery results suggested that the proposed portable electrochemical sensor had the potential to be applied to determine xylazine in beverage samples.

**Table 2:** Determination of Xylazine Levels in Beverage Samples (*n* = 3) with Recovery Values Using the Proposed Sensor

sample	spiked (μg mL^–1^)	found (*n* = 3) (μg mL^–1^)	% recovery (*n* = 3)
S1: Calpis Lacto	0.0	N.D.	
	5.0	4.2 ± 0.2	85 ± 3
	10.0	9.3 ± 0.2	92 ± 2
	20.0	17.8 ± 0.3	89 ± 1
	30.0	28.4 ± 0.2	94.7 ± 0.7
	40.0	39 ± 1	97 ± 3
S2: OISHI	0.0	N.D.	
	5.0	4.7 ± 0.1	94 ± 2
	10.0	8.9 ± 0.3	89 ± 3
	20.0	19.7 ± 0.6	99 ± 3
	30.0	29.2 ± 0.2	97.3 ± 0.5
	40.0	39.1 ± 0.7	98 ± 2
S3: Pepsi Max	0.0	N.D.	
	5.0	4.40 ± 0.06	84 ± 4
	10.0	8.8 ± 0.3	88 ± 3
	20.0	18.3 ± 0.7	91 ± 3
	30.0	28.2 ± 0.4	94 ± 1
	40.0	38.6 ± 0.6	97 ± 2
S4: Yanhee Vitamin water	0.0	N.D.	
	5.0	4.5 ± 0.2	89 ± 4
	10.0	9.1 ± 0.4	91 ± 4
	20.0	18.6 ± 0.9	93 ± 4
	30.0	27 ± 1	88 ± 4
	40.0	36.5 ± 0.9	91 ± 2
S5: Smirnoff Gold	0.0	N.D.	
	5.0	4.4 ± 0.2	88 ± 4
	10.0	9.3 ± 0.4	93 ± 4
	20.0	20.0 ± 0.2	100 ± 1
	30.0	30 ± 1	100 ± 4
	40.0	38.9 ± 0.7	97 ± 2
S6: Soda Rock Mountain	0.0	N.D.	
	5.0	4.4 ± 0.2	89 ± 3
	10.0	9.0 ± 0.3	90 ± 2
	20.0	20.1 ± 0.5	100 ± 3
	30.0	29.5 ± 0.3	98 ± 1
	40.0	39 ± 2	98 ± 5
S7: Jinro Chamisul Soju	0.0	N.D.	
	5.0	4.4 ± 0.1	87 ± 2
	10.0	9.4 ± 0.5	94 ± 4
	20.0	21.1 ± 0.5	105 ± 2
	30.0	28.5 ± 0.4	95 ± 1
	40.0	38.3 ± 0.7	96 ± 2

## Conclusions

We introduced a portable electrochemical xylazine sensor for on-site analysis that integrated a smartphone and a PANI-modified ePAD. The ePAD was successfully fabricated using craft printer/cutter and low-tack transfer tape to create the template mask for a screen-printing process. A uniform electrode pattern was coated on a chromatography paper with graphene ink. A large conductive surface area was produced, which was modified with coral-like PANI. A large number of adsorption sites were produced, facilitating the interaction between xylazine and the electrode surface. In the optimized condition, this portable sensor was used to directly detect xylazine by DPV. The sensor also exhibited good performances in terms of its linearity, detection limit, and reproducibility. Moreover, we successfully applied the easy-to-use, portable sensor to determine xylazine spiked in beverage samples. The sensor demonstrated its potential and suitability for use in real-case forensic scenarios: particularly where xylazine-spiked beverages are involved.

## Supporting Information Available

Components of the portable electrochemical sensor; body of the portable electrochemical device, monitor/software, and sensing part; linear relationships of current (*I*_p_) versus scan rate (υ), current (*I*_p_) versus square root of scan rate (υ^1/2^), and potential versus log of current; CV conditions; scan rates from 20 to 200 mV s^–1^ at PANI/ePAD in the BR buffer at pH 7.00 containing 10 μg mL^–1^ xylazine; and DPV response of possibly interfering species and other drugs on the peak current of 10 μg mL^–1^ xylazine (PDF)
