# Supplementary material for: Nanocoral-like Polyaniline-Modified Graphene-Based Electrochemical Paper-Based Analytical Device for a Portable Electrochemical Sensor for Xylazine Detection
Source: ACS Omega. 2022 Apr 12;7(16):13913–24. doi: 10.1021/acsomega.2c00295 (PMC9088932; doi:10.1021/acsomega.2c00295)
Supplement: Supplementary file 1 — ao2c00295_si_001.pdf [file ao2c00295_si_001.pdf]

Supporting Information

Nanocoral-like polyaniline modified graphene based electrochemical paper-based analytical device for portable electrochemical sensor for xylazine detection

Kasrin Saisahas[a], Asamee Soleh[b,c,d], Kiattisak Promsuwan[b,e, f], Jenjira Saichanapan[e,f], Apichai Phonchai[e,f], Nabeesathul Sumayya Mohamed Sadiq[a], Way Koon Teoh[a], Kah Haw Chang[a], Ahmad Fahmi Lim Abdullah[a**], Warakorn Limbut[b,c,e,f*]

[a] *Forensic Science Programme, School of Health Sciences, Universiti Sains Malaysia, 16150 Kubang Kerian, Kelantan, Malaysia*

[b] *Center of Excellence for Trace Analysis and Biosensors (TAB-CoE), Prince of Songkla University, Hat Yai, Songkhla 90110, Thailand.*

[c] *Center of Excellence for Innovation in Chemistry, Faculty of Science, Prince of Songkla University, Hat Yai, Songkhla 90110, Thailand*

[d] *Division of Physical Science, Faculty of Science, Prince of Songkla University, Hat Yai, Songkhla 90110, Thailand*

[e] *Division of Health and Applied Sciences, Faculty of Science, Prince of Songkla University, Hat Yai, Songkhla 90110, Thailand*

[f] *Forensic Science Innovation and Service Center, Prince of Songkla University, Hat Yai, Songkhla 90110, Thailand*

[*] *Corresponding author:* Division of Health and Applied Sciences, Faculty of Science, Prince of Songkla University, Hat Yai, Songkhla 90112, Thailand. Tel.: +66 74 288563; Fax: +66 74 446681

E-mail addresses: warakorn.l@psu.ac.th (W. Limbut)

[**] *Corresponding author:* Forensic Science Programme, School of Health Sciences, Universiti Sains Malaysia, 16150 Kubang Kerian, Kelantan, Malaysia. Tel.: +60 97 677596

E-mail addresses: fahmilim@usm.my (AFL Abdullah)

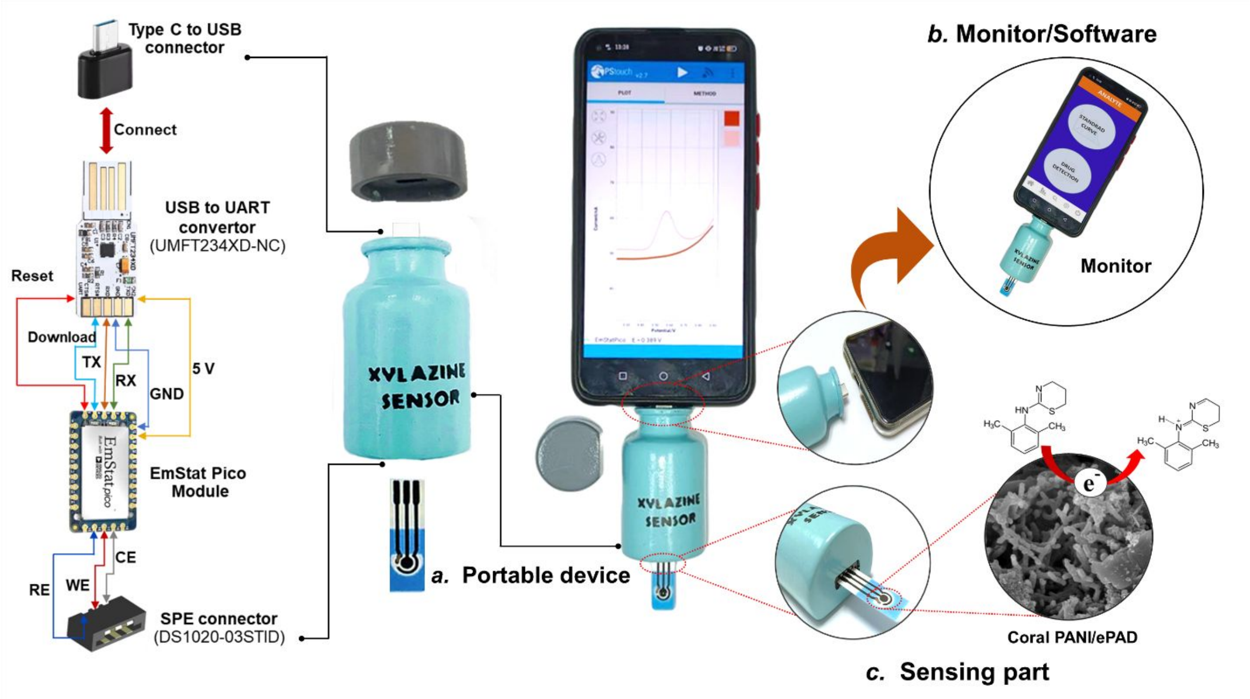

Figure S1. Components of the portable electrochemical sensor. (a) Body of the portable electrochemical device, (b) monitor/software, and (c) sensing part.

The portable electrochemical sensor for xylazine was designed in a pill bottle box case (Figure S1-a) using the program Solid work 2020 to design a three-dimensional (3D) model and manufacture with 3D Printer (GEEETECH E180 3D printer) by the fused deposition modeling (FDM) method using poly (lactic acid) (PLA) filament. The portable electrochemical device contains an Emstat Pico Module potentiostat (PalmSens, www.palmsens.com /product/oem-emstat-pico-module/). The Emstat Pico Module potentiostat will connected with two connectors, including a USB to UART convertor (UMFT234XD-NC) for connecting the device to a smartphone via a type-C USB connector. The other end of the Emstat Pico Module potentiostat will be connect with screen-printed electrode (SPE) connector (DS1020-03ST1D) for connecting the device to the ePAD electrode modified with coral-PANI (Figure S1-c). For an android

smartphone used to control the portable electrochemical sensor via the drug sensor application, which it was developed from PalmSens Software Development Kits (SDKs) for .NET (www.palmsens.com/oem/sdkdotnet/), which has two modes (i) standard curve for standard detection and (ii) drug detection for real-sample analysis (Figure S1-b)

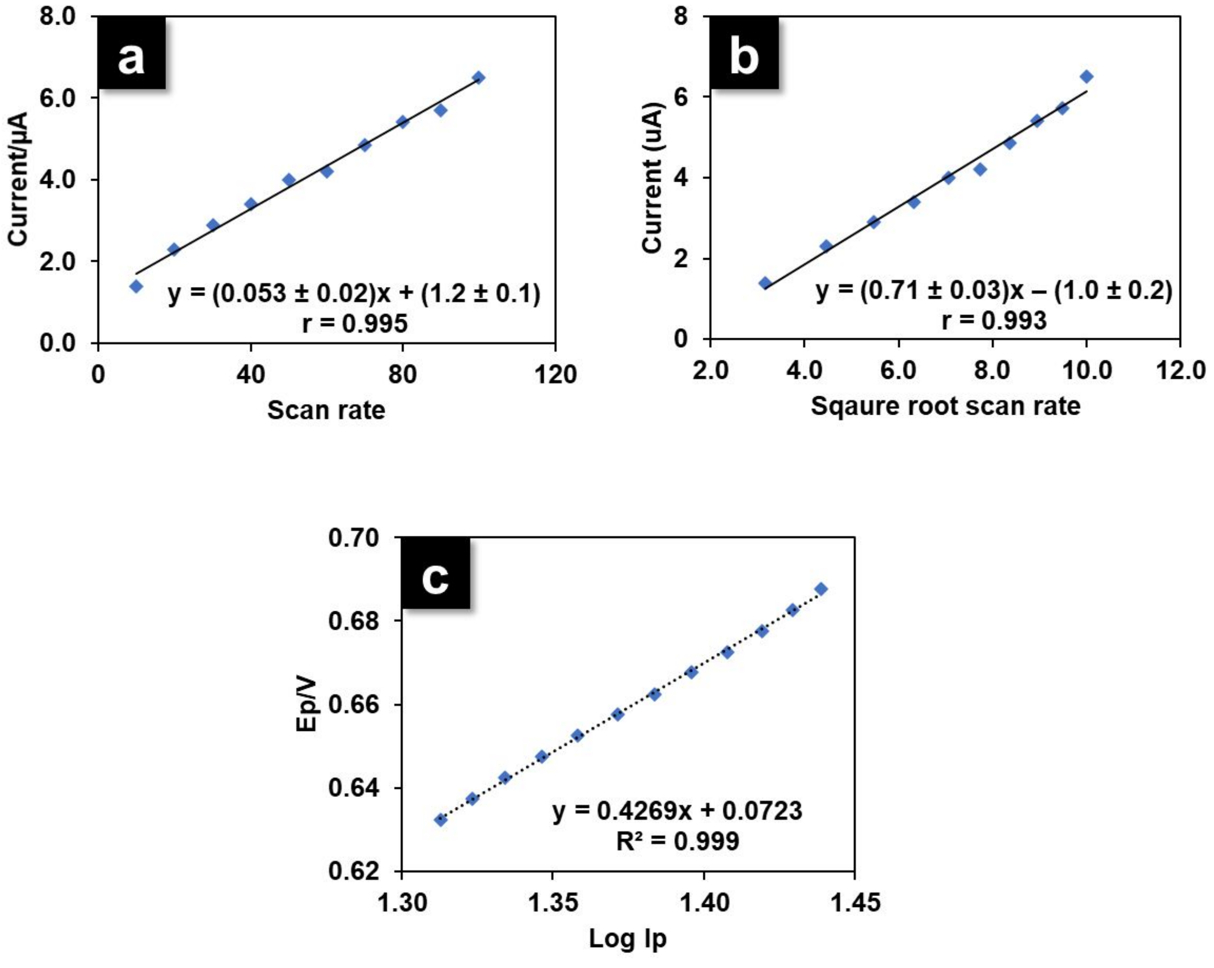

Figure S2. Linear relationships of *current* (I_p) *vs. scan rate* (v) (a), *current* (I_p) *vs. square root of scan rate* ($v^{1/2}$) (b), and potential vs log of current (c). (CV conditions; scan rates from 20-200 mV s^{-1} at the PANI/ePAD in BR buffer at pH 7.00 containing 10 µg mL^{-1} xylazine)

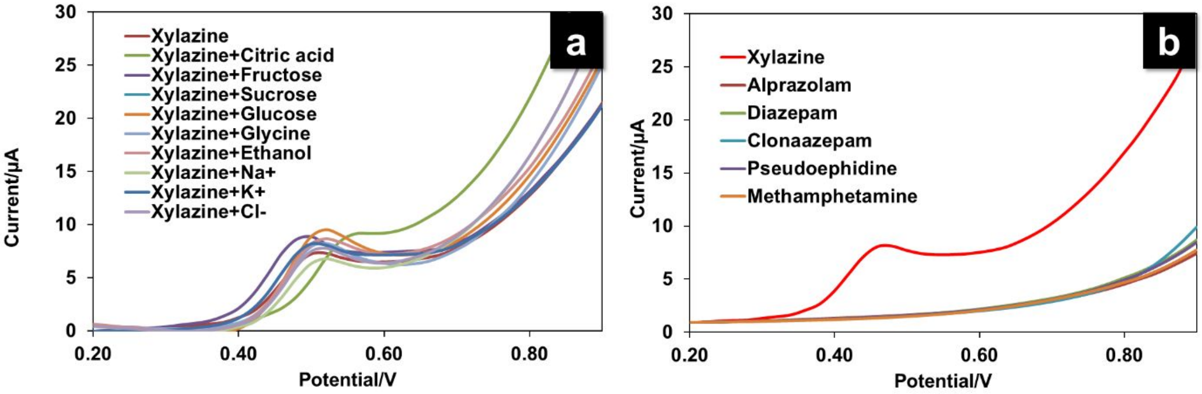

Figure S3. DPV response of possibly interfering species (a) and other drug (b) on the peak current of 10 μg mL^{-1} xylazine.
